# Supplementary figures and images for: Fine interaction profiling of VemP and mechanisms responsible for its translocation-coupled arrest-cancelation
Source: eLife. 2020 Dec 15;9:e62623. doi: 10.7554/eLife.62623 (PMC7793623; doi:10.7554/eLife.62623)

Figure 2 Source data

B

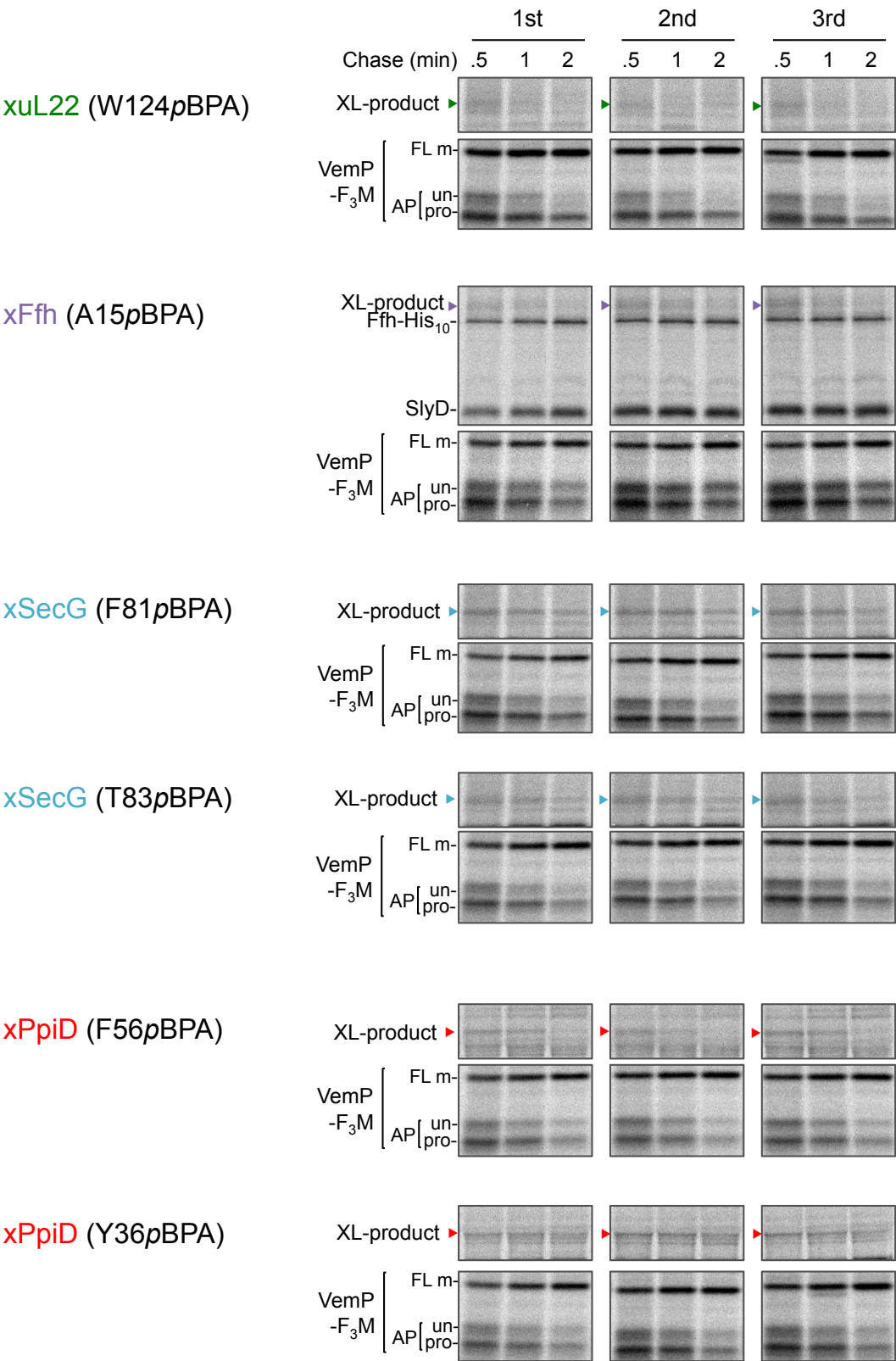

Figure 2 Source data

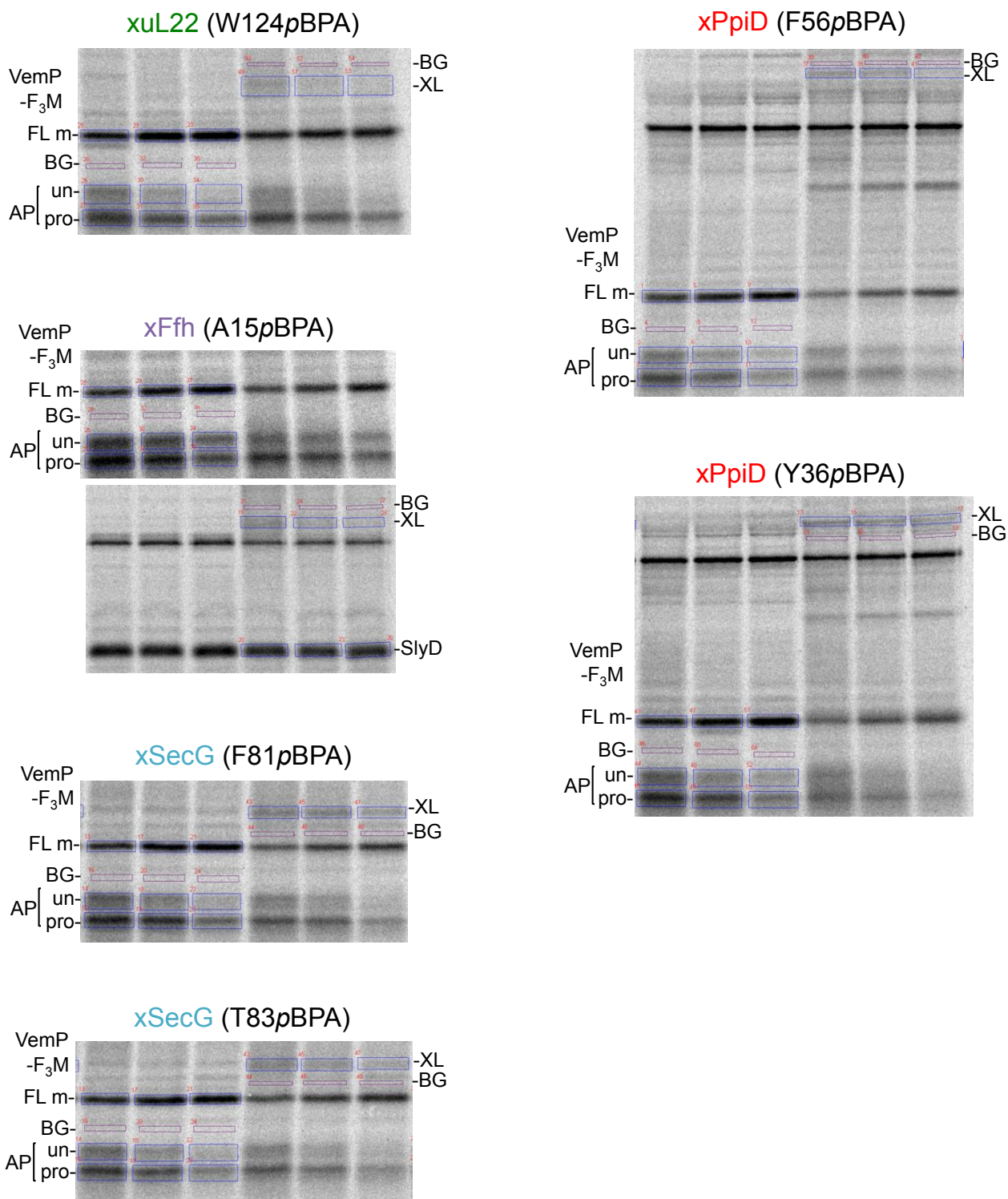

Supplement: Figure 2—source data 1. [file elife-62623-fig2-data1.zip › Figure 2/Figure 2-gel images.pdf]

Figure 2-supple 1 Source data

A

WT strain

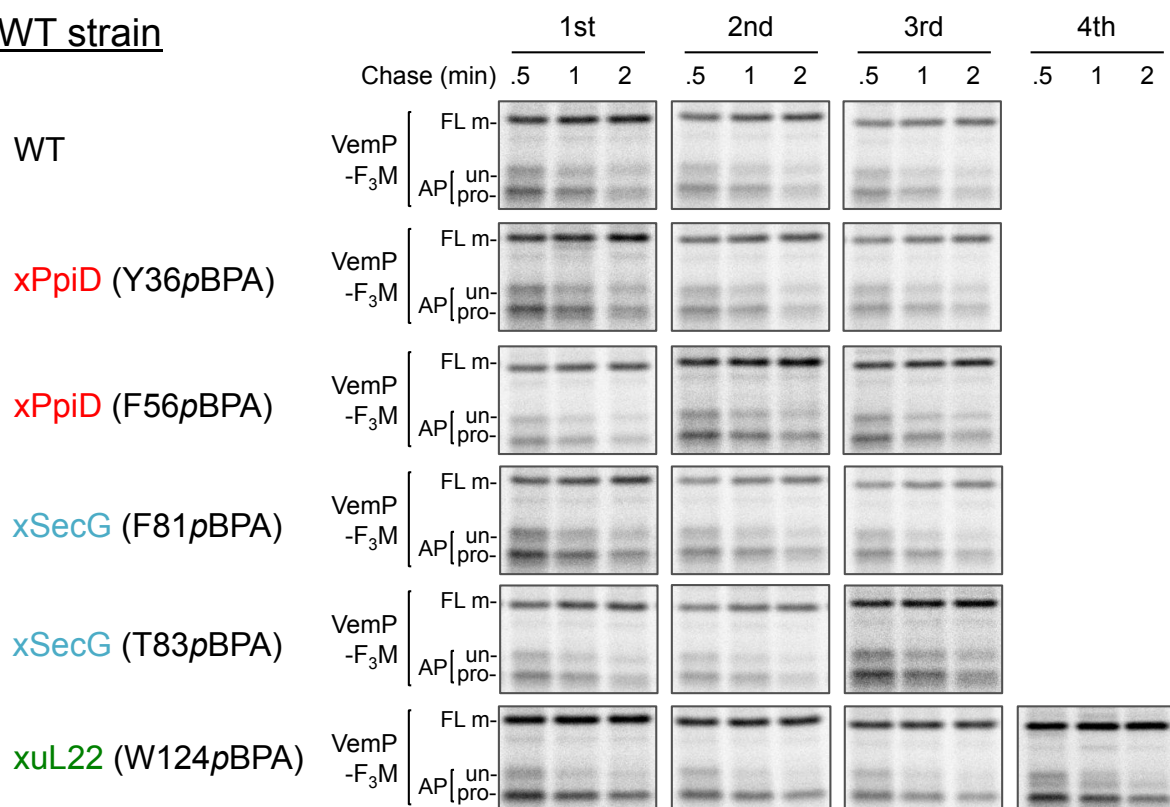

*ffh-his<sub>10</sub>* strain

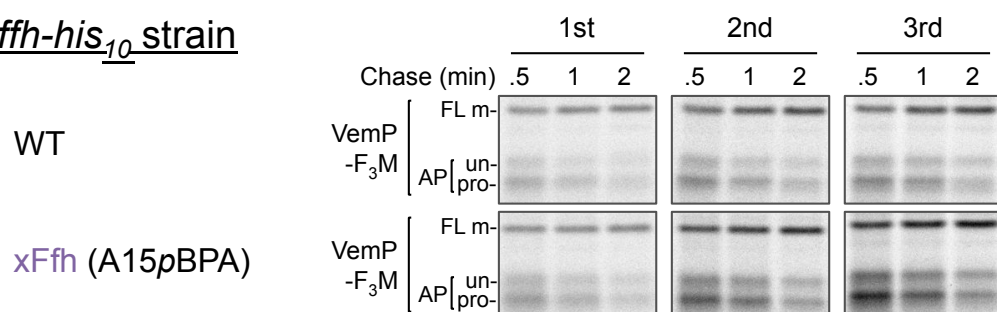

Supplement: Figure 2—figure supplement 1—source data 1. [file elife-62623-fig2-figsupp1-data1.zip › Figure 2-figure supplement 1/Figure 2-figure supplement 1-gel images.pdf]

Figure 2-supple 4 Source data

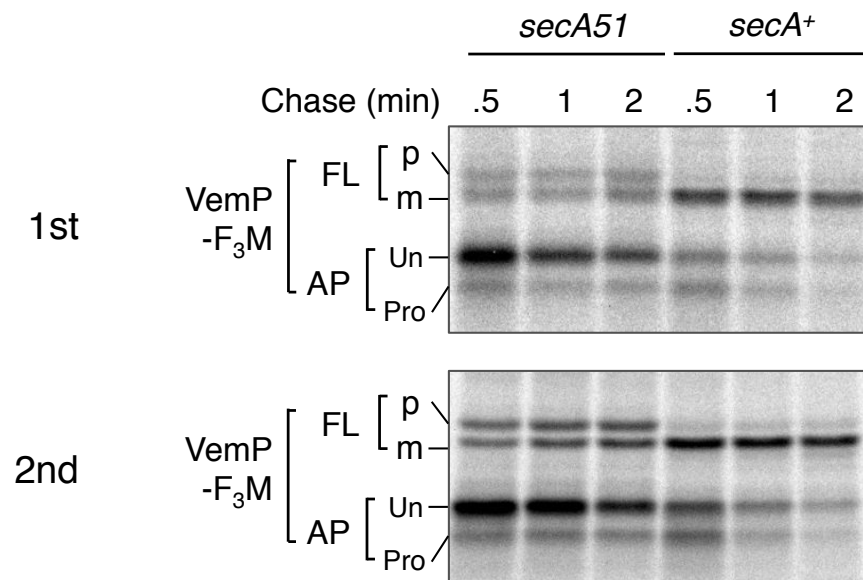

Supplement: Figure 2—figure supplement 3—source data 1. [file elife-62623-fig2-figsupp3-data1.zip › Figure 2-figure supplement 3/Figure 2-figure supplement 3-gel images.pdf]

Figure 2-supple 2 Source data

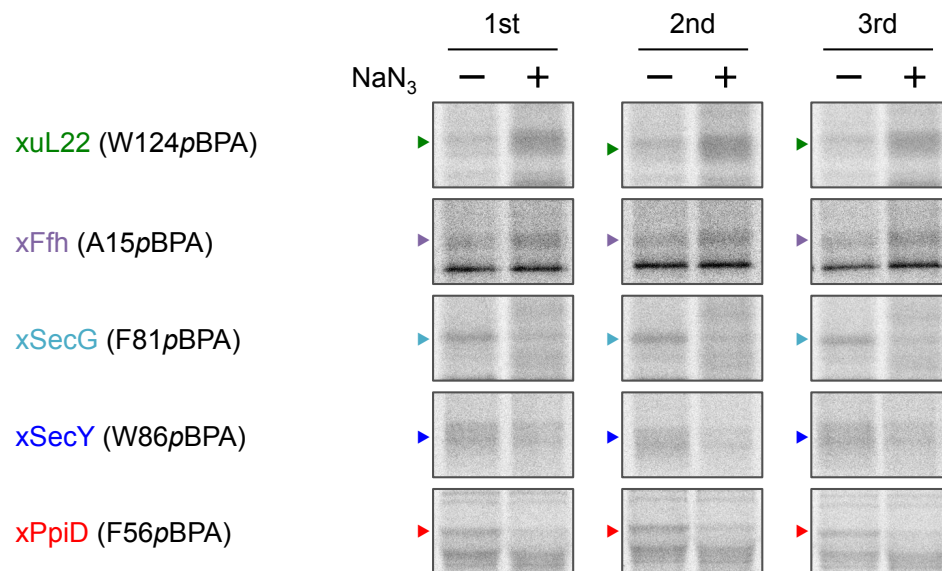

Supplement: Figure 2—figure supplement 4—source data 1. [file elife-62623-fig2-figsupp4-data1.zip › Figure 2-figure supplement 4/Figure 2-figure supplement 4-gel images.pdf]

Figure 3 Source data

A

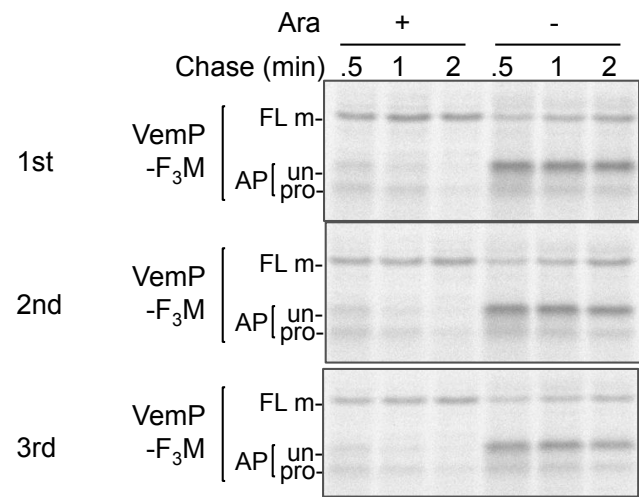

B

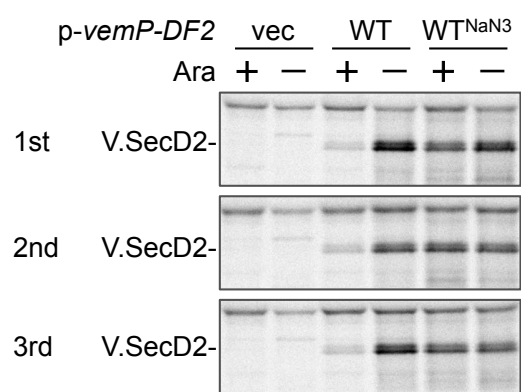

C

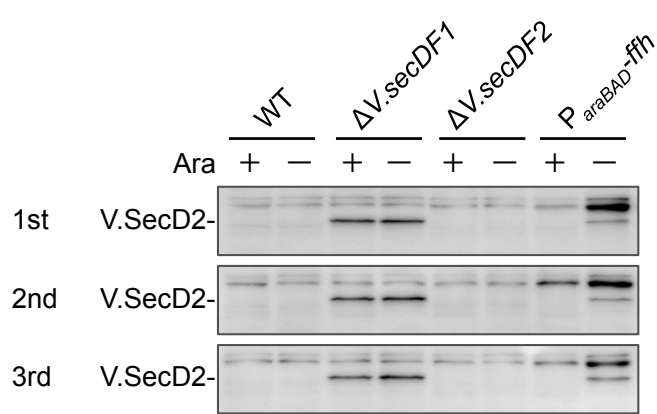

Supplement: Figure 3—source data 1. — (A–C) and quantified band intensity data for the pulse-chase experiments using the anti-VemP antibody (A), pulse-labeling experiments using the anti-SecD2 antibody (B) and immunoblotting with the anti-SecD2 antibody (C). [file elife-62623-fig3-data1.zip › Figure 3/Figure 3-gel images.pdf]

Figure 4 Source data

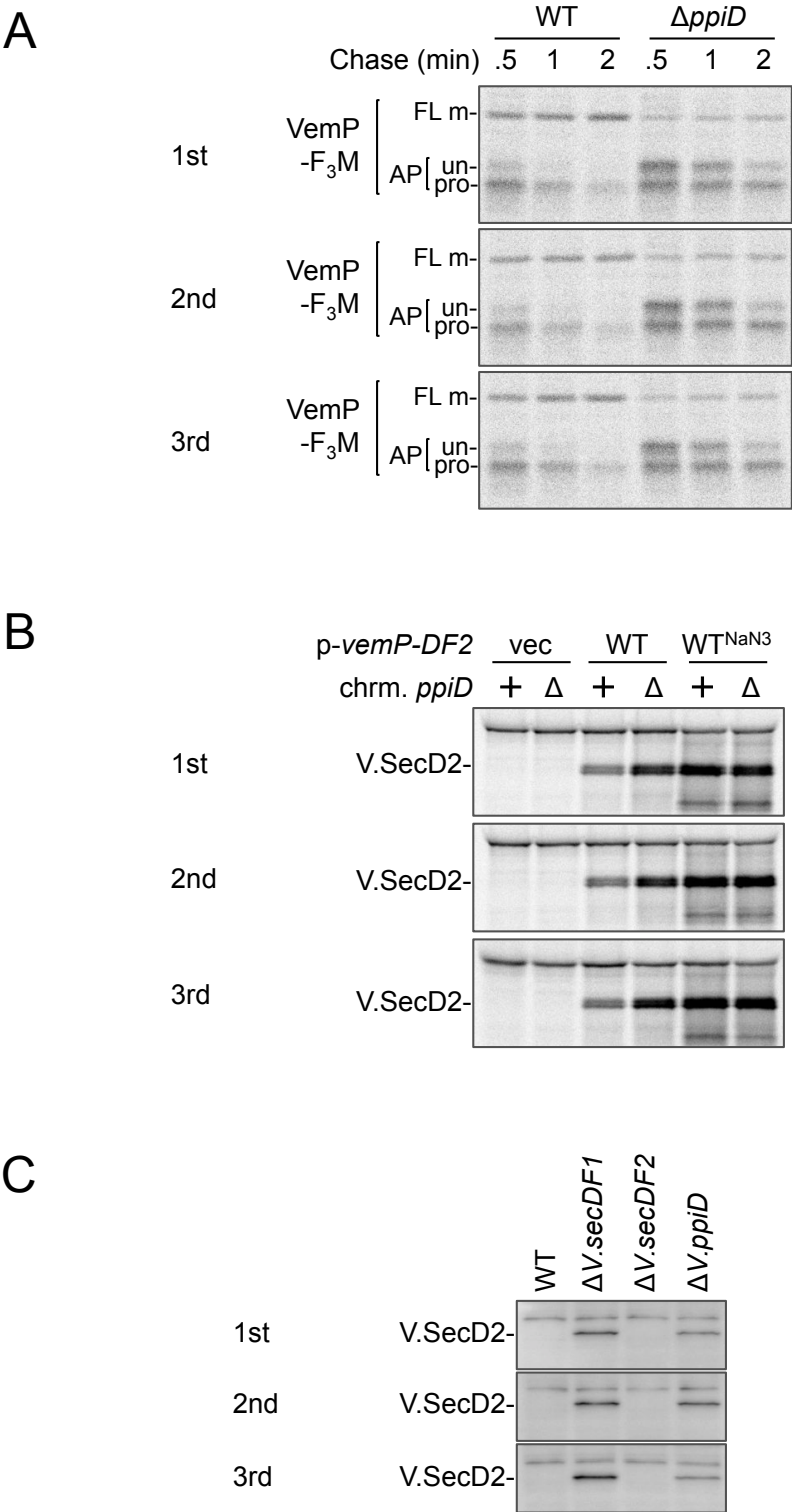

Supplement: Figure 4—source data 1. — (A–C) and quantified band intensity data for the pulse-chase experiments using the anti-VemP antibody (A), pulse-labeling experiments using the anti-SecD2 antibody (B) and immunoblotting with the anti-SecD2 antibody (C). [file elife-62623-fig4-data1.zip › Figure 4/Figure 4-gel images.pdf]

# Figure 5-supple 1 Source data

A

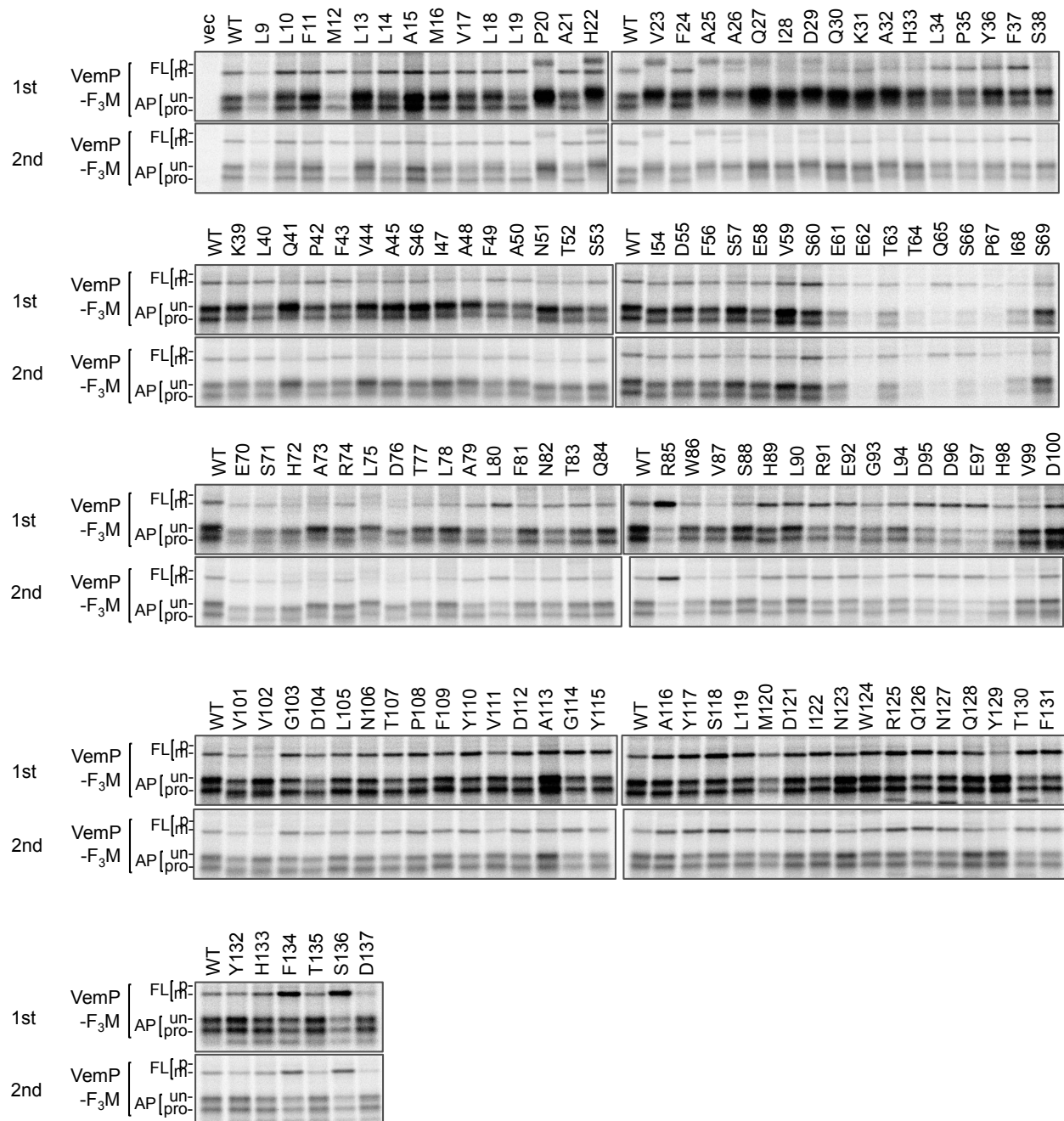

Figure 5-supple 1 Source data

B

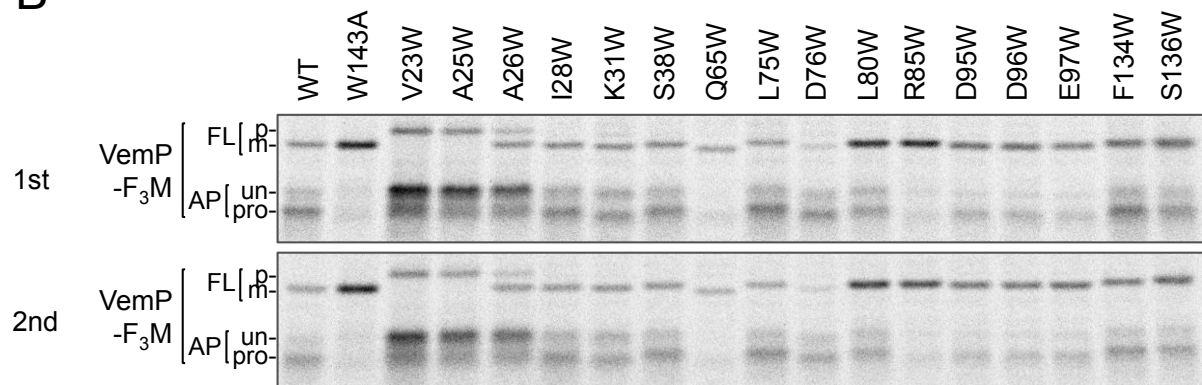

C

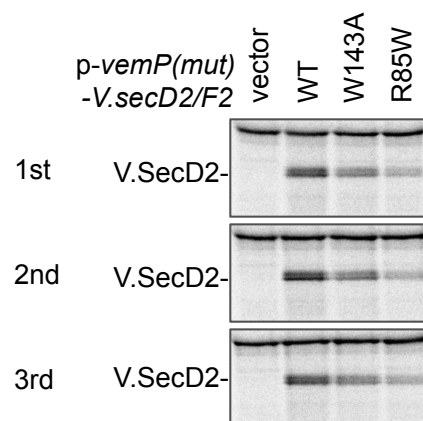

Supplement: Figure 5—figure supplement 1—source data 1. [file elife-62623-fig5-figsupp1-data1.zip › Figure 5-figure supplement 1/Figure 5-figure supplement 1-gel images.pdf]

Figure 5-supple 2 Source data

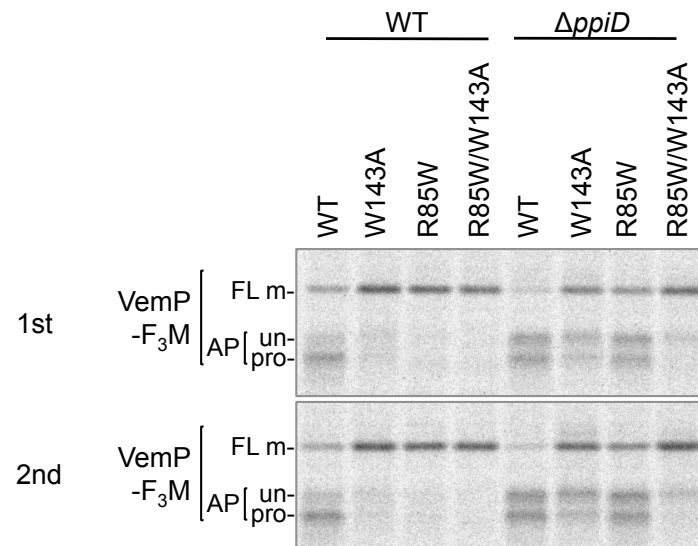

Supplement: Figure 5—figure supplement 2—source data 1. [file elife-62623-fig5-figsupp2-data1.zip › Figure 5-figure supplement 2/Figure 5-figure supplement 2-gel images.pdf]
