# Supplementary material for: Fine interaction profiling of VemP and mechanisms responsible for its translocation-coupled arrest-cancelation
Source: eLife. 2020 Dec 15;9:e62623. doi: 10.7554/eLife.62623 (PMC7793623; doi:10.7554/eLife.62623)
Supplement: Supplementary file 1. — Strains used in this study [file elife-62623-supp1.docx]

**Table S1. Strains used in this study.**

| (**A**) *E. coli* strains | |  |  |  |
| --- | --- | --- | --- | --- |
| Strain | Genotype |  |  | Reference |
| MC4100 | F^-^ *araD139* Δ*(argF-lac)U169 rpsL150 relA1 flbB5301 deoC1 ptsF25 rbsR* | | | (Silhavy et al., 1984) |
| CU141 | MC4100/F' *lacI*^q^ *lacZ*^+^, *Y*^+^, *A*^+^ | |  | (Akiyama et al., 1994) |
| HM1742 | CU141, *ara*^+^ |  |  | (Mori and Ito, 2006) |
| RM2831 | HM1742, *ffh-his_10_* | |  | This study |
| RM2834 | HM1742, *secY-his_10_* | |  | This study |
| RM2935 | HM1742, *yidC-his_10_ kan* | |  | This study |
| RM3032 | HM1742, *ppiD-his_10_ kan* | |  | This study |
| RM3122 | HM1742, Δ*ppiD*::*kan* | |  | This study |
| RM3124 | HM1742, Δ*secG*::*kan* | |  | This study |
| HM3271 | HM1742, *secD1 zaj-3053*::Tn*10* | |  | (Tsukazaki et al., 2011) |
| HM4108 | HM1742, *secY*^+^ *rpsE* *zhd-33*::Tn*10* | |  | (Mori et al., 2018) |
| HM4109 | HM1742, *secY24* *rpsE* *zhd-33*::Tn*10* | | | (Mori et al., 2018) |
| HM4798 | HM1742, Δ*secB*::FRT | | | This study |
| WAM121 | MC4100, *ara*^+^ *ffh1*::*kan* *attB*::R6K*ori* P*_ara_-ffh*^+^ *cat* | | | (de Gier et al., 1996) |
| DY330 | W3110, Δ*lacU169* *gal490* λ*cI857* Δ(*cro-bioA*) | | | (Yu et al., 2000) |
| RM2813 | DY330, *ffh-his_10_ kan* | |  | This study |
| RM2816 | DY330, *secY-his_10_ kan* | |  | This study |
| RM2934 | DY330, *yidC-his_10_ kan* | |  | This study |
| RM2999 | DY330, *ppiD-his_10_ kan* | |  | This study |
| KI269 | MC4100 *cya283*/ F' *lacI*^q^ *lacZ*^+^, *Y*^+^, *A*^+^ | |  | (Akiyama and Ito, 1985) |
| MM52 | MC4100 *secA51*(Ts) | |  | (Oliver and Beckwith, 1981) |
| AD76 | KI269 *secA51* | |  | This study |

| (**B**) *V. alginolyticus* strains | |  |  |  |
| --- | --- | --- | --- | --- |
| Strain | Genotype |  |  | Reference |
| VIO5 | 138-2 Rif^R^ Pof^+^ Laf^-^ | |  | (Okunishi et al., 1996) |
| NR23 | VIO5 Δ*V.secDF1*::*kan* | |  | (Ishii et al., 2015) |
| HM3740 | VIO5 Δ*V.secDF2*::*kan* | |  | (Ishii et al., 2015) |
| RMV2 | VIO5 Δ*V.ppiD* |  |  | This study |
| RMV7 | VIO5 P*_ara_-V.ffh* |  |  | This study |

**(References)**

Akiyama Y, Ito K. 1985. The SecY membrane component of the bacterial protein export machinery: analysis by new electrophoretic methods for integral membrane proteins. *EMBO J* **4**:3351–3356.

Akiyama Y, Ogura T, Ito K. 1994. Involvement of FtsH in protein assembly into and through the membrane. I. Mutations that reduce retention efficiency of a cytoplasmic reporter. *J Biol Chem* **269**:5218–5224.

de Gier JW, Mansournia P, Valent QA, Phillips GJ, Luirink J, von Heijne G. 1996. Assembly of a cytoplasmic membrane protein in *Escherichia coli* is dependent on the signal recognition particle. *FEBS Lett* **399**:307–309. doi:10.1016/s0014-5793(96)01354-3

Ishii E, Chiba S, Hashimoto N, Kojima S, Homma M, Ito K, Akiyama Y, Mori H. 2015. Nascent chain-monitored remodeling of the Sec machinery for salinity adaptation of marine bacteria. *Proc Natl Acad Sci U S A* **112**:E5513-22. doi:10.1073/pnas.1513001112

Mori H, Ito K. 2006. The long α-helix of SecA is important for the ATPase coupling of translocation. *J Biol Chem* **281**:36249–36256. doi:10.1074/jbc.M606906200

Mori H, Sakashita S, Ito J, Ishii E, Akiyama Y. 2018. Identification and characterization of a translation arrest motif in VemP by systematic mutational analysis. *J Biol Chem* **293**:2915–2926. doi:10.1074/jbc.M117.816561

Okunishi I, Kawagishi I, Homma M. 1996. Cloning and characterization of *motY*, a gene coding for a component of the sodium-driven flagellar motor in *Vibrio alginolyticus*. *J Bacteriol* **178**:2409–2415. doi:10.1128/jb.178.8.2409-2415.1996

Oliver DB, Beckwith J. 1981. E. coli mutant pleiotropically defective in the export of secreted proteins. *Cell* **25**:765–772. doi:10.1016/0092-8674(81)90184-7

Silhavy TJ, Berman ML, Enquist LW, editors. 1984. Experiments With Gene Fusions. New York: Cold Spring Harbor Laboratory Press.

Tsukazaki T, Mori H, Echizen Y, Ishitani R, Fukai S, Tanaka T, Perederina A, Vassylyev DG, Kohno T, Maturana AD, Ito K, Nureki O. 2011. Structure and function of a membrane component SecDF that enhances protein export. *Nature* **474**:235–238. doi:10.1038/nature09980

Yu D, Ellis HM, Lee EC, Jenkins NA, Copeland NG, Court DL. 2000. An efficient recombination system for chromosome engineering in *Escherichia coli.* *Proc Natl Acad Sci U S A* **97**:5978–5983. doi:10.1073/pnas.100127597
