# Supplementary material for: Fine interaction profiling of VemP and mechanisms responsible for its translocation-coupled arrest-cancelation
Source: eLife. 2020 Dec 15;9:e62623. doi: 10.7554/eLife.62623 (PMC7793623; doi:10.7554/eLife.62623)
Supplement: Supplementary file 2. — Plasmids used in this study [file elife-62623-supp2.docx]

**Table S2. Plasmids used in this study.**

| Plasmid | Vector | Gene and description | Reference or source | Relevant experiment |
| --- | --- | --- | --- | --- |
| pEVOL-pBpF |  | p15A-derivative encoding an evolved *M. jannaschii* aminoacyl-tRNA synthetase/ suppressor tRNA pair for incorporation of *p*BPA; Cm^R^ | (Young et al., 2010) | Figs. 1B-F, 2A, Fig. 1-figure supplements 1, 2, 3, 4, 5, Fig. 2-figure supplements 1, 3 |
| pHM1021 |  | Expression vector; P*_lac_*, Amp^R^ | (Ishii et al., 2015) |  |
| pTS47 | pHM1021 | *vemP-3xflag-myc* *NcoI site is absent | (Mori et al., 2018) | Figs. 3A, Fig. 3-figure supplement 1 |
| pTS48 | pHM1021 | *vemP-3xflag-myc* | This study | Figs. 1C-F, 4A, B, 5A, C, Fig. 1-figure supplements 2, 3, 4, 5, Fig. 2-figure supplements 1, 3, 4, Fig. 5-figure supplement 1, 2 |
| pRM370 | pHM1021 | *vemP(F11amb)-3xflag-myc* | This study | Fig. 1-figure supplement 4 |
| pRM549 | pHM1021 | *vemP(L13amb)-3xflag-myc* | This study |  |
| pRM550 | pHM1021 | *vemP(A15amb)-3xflag-myc* | This study | Figs. 1E, 2A, Fig. 1-figure supplements 4, 5, Fig. 2-figure supplements 1, 4 |
| pRM371 | pHM1021 | *vemP(M16amb)-3xflag-myc* | This study |  |
| pRM551 | pHM1021 | *vemP(L18amb)-3xflag-myc* | This study | Fig. 1-figure supplement 4 |
| pRM372 | pHM1021 | *vemP(A21amb)-3xflag-myc* | This study |  |
| pRM373 | pHM1021 | *vemP(F24amb)-3xflag-myc* | This study | Fig. 1-figure supplement 4 |
| pRM348 | pHM1021 | *vemP(K31amb)-3xflag-myc* | This study |  |
| pRM349 | pHM1021 | *vemP(Y36amb)-3xflag-myc* | This study | Figs. 1F, 2A, Fig. 1-figure supplements 3, 4, Fig. 2-figure supplement 1 |
| pRM552 | pHM1021 | *vemP(L40amb)-3xflag-myc* | This study | Fig. 1-figure supplement 4 |
| pRM350 | pHM1021 | *vemP(Q41amb)-3xflag-myc* | This study |  |
| pRM351 | pHM1021 | *vemP(S46amb)-3xflag-myc* | This study | Figs. 1F, Fig. 1-figure supplement 4 |
| pRM553 | pHM1021 | *vemP(F49amb)-3xflag-myc* | This study | Figs. 1F, Fig. 1-figure supplement 4 |
| pRM352 | pHM1021 | *vemP(N51amb)-3xflag-myc* | This study |  |
| pRM353 | pHM1021 | *vemP(F56amb)-3xflag-myc* | This study | Figs. 1E, Fig. 1-figure supplements3, 4, 5, Fig. 2-figure supplements 1, 4 |
| pRM554 | pHM1021 | *vemP(S60amb)-3xflag-myc* | This study | Figs. 1F, Fig. 1-figure supplement 4 |
| pRM354 | pHM1021 | *vemP(E61amb)-3xflag-myc* | This study |  |
| pRM355 | pHM1021 | *vemP(S66amb)-3xflag-myc* | This study |  |
| pRM356 | pHM1021 | *vemP(S71amb)-3xflag-myc* | This study |  |
| pRM595 | pHM1021 | *vemP(R74amb)-3xflag-myc* | This study | Fig. 1D |
| pRM357 | pHM1021 | *vemP(D76amb)-3xflag-myc* | This study | Figs. 1D, Fig. 1-figure supplement 4 |
| pRM586 | pHM1021 | *vemP(L78amb)-3xflag-myc* | This study | Fig. 1D |
| pRM358 | pHM1021 | *vemP(F81amb)-3xflag-myc* | This study | Figs. 1C, 2A, Fig. 1-figure supplements 3, 4, 5, Fig. 2-figure supplements 1, 4 |
| pRM597 | pHM1021 | *vemP(N82amb)-3xflag-myc* | This study | Figs. 1D, Fig.2-figure supplement 1 |
| pRM555 | pHM1021 | *vemP(T83amb)-3xflag-myc* | This study | Figs. 1D, 2A, Fig. 2-figure supplement 1 |
| pRM359 | pHM1021 | *vemP(W86amb)-3xflag-myc* | This study | Figs. 1D, Fig. 1-figure supplements 4, 5, Fig.2-figure supplement 4 |
| pRM360 | pHM1021 | *vemP(R91amb)-3xflag-myc* | This study | Fig. 1-figure supplement 4 |
| pRM361 | pHM1021 | *vemP(D96amb)3xflag-myc* | This study |  |
| pRM362 | pHM1021 | *vemP(V101amb)-3xflag-myc* | This study | Fig. 1-figure supplement 4 |
| pRM363 | pHM1021 | *vemP(N106amb)-3xflag-myc* | This study | Fig. 1-figure supplement 4 |
| pRM364 | pHM1021 | *vemP(V111amb)-3xflag-myc* | This study |  |
| pRM602 | pHM1021 | *vemP(A113amb)-3xflag-myc* | This study | Fig. 1-figure supplement 4 |
| pRM365 | pHM1021 | *vemP(A116amb)-3xflag-myc* | This study |  |
| pRM556 | pHM1021 | *vemP(Y117amb)-3xflag-myc* | This study | Fig. 1-figure supplement 4 |
| pRM366 | pHM1021 | *vemP(D121amb)-3xflag-myc* | This study |  |
| pRM557 | pHM1021 | *vemP(W124amb)-3xflag-myc* | This study | Figs. 2A, Fig. 1-figure supplements 4, 5, Fig. 2-figure supplements 1, 4 |
| pRM367 | pHM1021 | *vemP(Q126amb)-3xflag-myc* | This study |  |
| pRM368 | pHM1021 | *vemP(F131amb)-3xflag-myc* | This study |  |
| pRM369 | pHM1021 | *vemP(S136amb)-3xflag-myc* | This study |  |
| pTS50 | pHM1021 | *vemP(W143A)-3xflag-myc* | This study | Figs. 5A, B, Fig. 5-figure supplements 1, 2 |
| pRM542 | pHM1021 | *vemP(R85W)-3xflag-myc* | This study | Figs. 5A, B, Fig. 5-figure supplements 1, 2 |
| pRM543 | pHM1021 | *vemP(R85K)-3xflag-myc* | This study | Fig. 5A |
| pRM544 | pHM1021 | *vemP(R85E)-3xflag-myc* | This study | Fig. 5A |
| pRM545 | pHM1021 | *vemP(R85G)-3xflag-myc* | This study | Fig. 5A |
| pRM546 | pHM1021 | *vemP(R85A)-3xflag-myc* | This study | Fig. 5A |
| pRM547 | pHM1021 | *vemP(R85L)-3xflag-myc* | This study | Fig. 5A |
| pRM603 | pHM1021 | *vemP(V23W)-3xflag-myc* | This study | Fig. 5-figure supplement 1 |
| pRM604 | pHM1021 | *vemP(A25W)-3xflag-myc* | This study | Fig. 5-figure supplement 1 |
| pRM605 | pHM1021 | *vemP(A26W)-3xflag-myc* | This study | Fig. 5-figure supplement 1 |
| pRM612 | pHM1021 | *vemP(I28W)-3xflag-myc* | This study | Fig. 5-figure supplement 1 |
| pRM606 | pHM1021 | *vemP(K31W)-3xflag-myc* | This study | Fig. 5-figure supplement 1 |
| pRM613 | pHM1021 | *vemP(S38W)-3xflag-myc* | This study | Fig. 5-figure supplement 1 |
| pRM614 | pHM1021 | *vemP(Q65W)-3xflag-myc* | This study | Fig. 5-figure supplement 1 |
| pRM615 | pHM1021 | *vemP(L75W)-3xflag-myc* | This study | Fig. 5-figure supplement 1 |
| pRM616 | pHM1021 | *vemP(D76W)-3xflag-myc* | This study | Fig. 5-figure supplement 1 |
| pRM617 | pHM1021 | *vemP(L80W)-3xflag-myc* | This study | Fig. 5-figure supplement 1 |
| pRM607 | pHM1021 | *vemP(D95W)-3xflag-myc* | This study | Fig. 5-figure supplement 1 |
| pRM611 | pHM1021 | *vemP(D96W)-3xflag-myc* | This study | Fig. 5-figure supplement 1 |
| pRM608 | pHM1021 | *vemP(E97W)-3xflag-myc* | This study | Fig. 5-figure supplement 1 |
| pRM609 | pHM1021 | *vemP(F134W)-3xflag-myc* | This study | Fig. 5-figure supplement 1 |
| pRM610 | pHM1021 | *vemP(S136W)-3xflag-myc* | This study | Fig. 5-figure supplement 1 |
| pRM847 | pHM1021 | *vemP(R85W, W143A)-3xflag-myc* | This study | Fig.5- Fig. 5-figure supplement 2 |
| pHM1202 | pHM1021 | *vemP(F.S.)-3xflag-myc* | (Mori et al., 2018) | Fig. 5C |
| pRM848 | pHM1021 | *vemP(F.S., R85W)-3xflag-myc* | This study | Fig. 5C |
| pRM663 | pHM1021 | *vemP-V.secD2/F2* | This study | Figs. 3B, 4B, Fig. 5-figure supplement 1 |
| pRM666 | pHM1021 | *vemP(W143A)-V.secD2/F2* | This study | Fig. 5-figure supplement 1 |
| pRM667 | pHM1021 | *vemP(R85W)-V.secD2/F2* | This study | Fig. 5-figure supplement 1 |
| pTV118N |  | Expression vector; P*_lac_*, Amp^R^ | Takara Bio |  |
| pRM374 | pTV118N | *vemP-3xflag-myc* | This study | Fig. 1-figure supplement 1 |
| pRM409 | pTV118N | *vemP(L9amb)-3xflag-myc* | This study | Fig. 1-figure supplement 1 |
| pRM503 | pTV118N | *vemP(L10amb)-3xflag-myc* | This study | Fig. 1-figure supplement 1 |
| pRM382 | pTV118N | *vemP(F11amb)-3xflag-myc* | This study | Fig. 1-figure supplement 1 |
| pRM435 | pTV118N | *vemP(M12amb)-3xflag-myc* | This study | Fig. 1-figure supplement 1 |
| pRM461 | pTV118N | *vemP(L13amb)-3xflag-myc* | This study | Fig. 1-figure supplement 1 |
| pRM410 | pTV118N | *vemP(L14amb)-3xflag-myc* | This study | Fig. 1-figure supplement 1 |
| pRM503 | pTV118N | *vemP(A15amb)-3xflag-myc* | This study | Fig. 1-figure supplements 1, 2 |
| pRM387 | pTV118N | *vemP(M16amb)-3xflag-myc* | This study | Fig. 1-figure supplement 1 |
| pRM436 | pTV118N | *vemP(V17amb)-3xflag-myc* | This study | Fig. 1-figure supplement 1 |
| pRM462 | pTV118N | *vemP(L18amb)-3xflag-myc* | This study | Fig. 1-figure supplement 1 |
| pRM411 | pTV118N | *vemP(L19amb)-3xflag-myc* | This study | Fig. 1-figure supplement 1 |
| pRM486 | pTV118N | *vemP(P20amb)-3xflag-myc* | This study | Fig. 1-figure supplement 1 |
| pRM388 | pTV118N | *vemP(A21amb)-3xflag-myc* | This study | Fig. 1-figure supplement 1 |
| pRM437 | pTV118N | *vemP(H22amb)-3xflag-myc* | This study | Fig. 1-figure supplement 1 |
| pRM463 | pTV118N | *vemP(V23amb)-3xflag-myc* | This study | Fig. 1-figure supplement 1 |
| pRM389 | pTV118N | *vemP(F24amb)-3xflag-myc* | This study | Fig. 1-figure supplement 1 |
| pRM505 | pTV118N | *vemP(A25amb)-3xflag-myc* | This study | Fig. 1-figure supplement 1 |
| pRM412 | pTV118N | *vemP(A26amb)-3xflag-myc* | This study | Fig. 1-figure supplement 1 |
| pRM438 | pTV118N | *vemP(Q27amb)-3xflag-myc* | This study | Fig. 1-figure supplement 1 |
| pRM464 | pTV118N | *vemP(I28amb)-3xflag-myc* | This study | Fig. 1-figure supplement 1 |
| pRM429 | pTV118N | *vemP(D29amb)-3xflag-myc* | This study | Fig. 1-figure supplement 1 |
| pRM487 | pTV118N | *vemP(Q30amb)-3xflag-myc* | This study | Fig. 1-figure supplement 1 |
| pRM383 | pTV118N | *vemP(K31amb)-3xflag-myc* | This study | Fig. 1-figure supplement 1 |
| pRM439 | pTV118N | *vemP(A32amb)-3xflag-myc* | This study | Fig. 1-figure supplement 1 |
| pRM465 | pTV118N | *vemP(H33amb)-3xflag-myc* | This study | Fig. 1-figure supplement 1 |
| pRM413 | pTV118N | *vemP(L34amb)-3xflag-myc* | This study | Fig. 1-figure supplement 1 |
| pRM488 | pTV118N | *vemP(P35amb)-3xflag-myc* | This study | Fig. 1-figure supplement 1 |
| pRM384 | pTV118N | *vemP(Y36amb)-3xflag-myc* | This study | Fig. 1-figure supplement 1 |
| pRM440 | pTV118N | *vemP(F37amb)-3xflag-myc* | This study | Fig. 1-figure supplement 1 |
| pRM480 | pTV118N | *vemP(S38amb)-3xflag-myc* | This study | Fig. 1-figure supplement 1 |
| pRM414 | pTV118N | *vemP(K39amb)-3xflag-myc* | This study | Fig. 1-figure supplement 1 |
| pRM489 | pTV118N | *vemP(L40amb)-3xflag-myc* | This study | Fig. 1-figure supplements 1, 2 |
| pRM390 | pTV118N | *vemP(Q41amb)-3xflag-myc* | This study | Fig. 1-figure supplement 1 |
| pRM441 | pTV118N | *vemP(P42amb)-3xflag-myc* | This study | Fig. 1-figure supplement 1 |
| pRM466 | pTV118N | *vemP(F43amb)-3xflag-myc* | This study | Fig. 1-figure supplement 1 |
| pRM430 | pTV118N | *vemP(V44amb)-3xflag-myc* | This study | Fig. 1-figure supplement 1 |
| pRM490 | pTV118N | *vemP(A45amb)-3xflag-myc* | This study | Fig. 1-figure supplement 1 |
| pRM391 | pTV118N | *vemP(S46amb)-3xflag-myc* | This study | Fig. 1-figure supplement 1 |
| pRM442 | pTV118N | *vemP(I47amb)-3xflag-myc* | This study | Fig. 1-figure supplement 1 |
| pRM467 | pTV118N | *vemP(A48amb)-3xflag-myc* | This study | Fig. 1-figure supplement 1 |
| pRM431 | pTV118N | *vemP(F49amb)-3xflag-myc* | This study | Fig. 1-figure supplement 1 |
| pRM506 | pTV118N | *vemP(A50amb)-3xflag-myc* | This study | Fig. 1-figure supplement 1 |
| pRM392 | pTV118N | *vemP(N51amb)-3xflag-myc* | This study | Fig. 1-figure supplement 1 |
| pRM443 | pTV118N | *vemP(T52amb)-3xflag-myc* | This study | Fig. 1-figure supplement 1 |
| pRM481 | pTV118N | *vemP(S53amb)-3xflag-myc* | This study | Fig. 1-figure supplement 1 |
| pRM415 | pTV118N | *vemP(I54amb)-3xflag-myc* | This study | Fig. 1-figure supplement 1 |
| pRM507 | pTV118N | *vemP(D55amb)-3xflag-myc* | This study | Fig. 1-figure supplement 1 |
| pRM385 | pTV118N | *vemP(F56amb)-3xflag-myc* | This study | Fig. 1-figure supplements 1, 2 |
| pRM444 | pTV118N | *vemP(S57amb)-3xflag-myc* | This study | Fig. 1-figure supplement 1 |
| pRM482 | pTV118N | *vemP(E58amb)-3xflag-myc* | This study | Fig. 1-figure supplement 1 |
| pRM432 | pTV118N | *vemP(V59amb)-3xflag-myc* | This study | Fig. 1-figure supplement 1 |
| pRM491 | pTV118N | *vemP(S60amb)-3xflag-myc* | This study | Fig. 1-figure supplement 1 |
| pRM393 | pTV118N | *vemP(E61amb)-3xflag-myc* | This study | Fig. 1-figure supplement 1 |
| pRM458 | pTV118N | *vemP(E62amb)-3xflag-myc* | This study | Fig. 1-figure supplement 1 |
| pRM468 | pTV118N | *vemP(T63amb)-3xflag-myc* | This study | Fig. 1-figure supplement 1 |
| pRM416 | pTV118N | *vemP(T64amb)-3xflag-myc* | This study | Fig. 1-figure supplement 1 |
| pRM492 | pTV118N | *vemP(Q65amb)-3xflag-myc* | This study | Fig. 1-figure supplement 1 |
| pRM394 | pTV118N | *vemP(S66amb)-3xflag-myc* | This study | Fig. 1-figure supplement 1 |
| pRM445 | pTV118N | *vemP(P67amb)-3xflag-myc* | This study | Fig. 1-figure supplement 1 |
| pRM469 | pTV118N | *vemP(I68amb)-3xflag-myc* | This study | Fig. 1-figure supplement 1 |
| pRM417 | pTV118N | *vemP(S69amb)-3xflag-myc* | This study | Fig. 1-figure supplement 1 |
| pRM508 | pTV118N | *vemP(E70amb)-3xflag-myc* | This study | Fig. 1-figure supplement 1 |
| pRM395 | pTV118N | *vemP(S71amb)-3xflag-myc* | This study | Fig. 1-figure supplement 1 |
| pRM459 | pTV118N | *vemP(H72amb)-3xflag-myc* | This study | Fig. 1-figure supplement 1 |
| pRM483 | pTV118N | *vemP(A73amb)-3xflag-myc* | This study | Fig. 1-figure supplement 1 |
| pRM418 | pTV118N | *vemP(R74amb)-3xflag-myc* | This study | Fig. 1-figure supplement 1 |
| pRM493 | pTV118N | *vemP(L75amb)-3xflag-myc* | This study | Fig. 1-figure supplement 1 |
| pRM396 | pTV118N | *vemP(D76amb)-3xflag-myc* | This study | Fig. 1-figure supplement 1 |
| pRM446 | pTV118N | *vemP(T77amb)-3xflag-myc* | This study | Fig. 1-figure supplement 1 |
| pRM484 | pTV118N | *vemP(L78amb)-3xflag-myc* | This study | Fig. 1-figure supplement 1 |
| pRM433 | pTV118N | *vemP(A79amb)-3xflag-myc* | This study | Fig. 1-figure supplement 1 |
| pRM494 | pTV118N | *vemP(L80amb)-3xflag-myc* | This study | Fig. 1-figure supplement 1 |
| pRM397 | pTV118N | *vemP(F81amb)-3xflag-myc* | This study | Fig. 1-figure supplement 1 |
| pRM447 | pTV118N | *vemP(N82amb)-3xflag-myc* | This study | Fig. 1-figure supplement 1 |
| pRM470 | pTV118N | *vemP(T83amb)-3xflag-myc* | This study | Fig. 1-figure supplement 1 |
| pRM419 | pTV118N | *vemP(Q84amb)-3xflag-myc* | This study | Fig. 1-figure supplement 1 |
| pRM509 | pTV118N | *vemP(R85amb)-3xflag-myc* | This study | Fig. 1-figure supplement 1 |
| pRM398 | pTV118N | *vemP(W86amb)-3xflag-myc* | This study | Fig. 1-figure supplement 1 |
| pRM460 | pTV118N | *vemP(V87amb)-3xflag-myc* | This study | Fig. 1-figure supplement 1 |
| pRM471 | pTV118N | *vemP(S88amb)-3xflag-myc* | This study | Fig. 1-figure supplement 1 |
| pRM420 | pTV118N | *vemP(H89amb)-3xflag-myc* | This study | Fig. 1-figure supplement 1 |
| pRM495 | pTV118N | *vemP(L90amb)-3xflag-myc* | This study | Fig. 1-figure supplement 1 |
| pRM399 | pTV118N | *vemP(R91amb)-3xflag-myc* | This study | Fig. 1-figure supplement 1 |
| pRM448 | pTV118N | *vemP(E92amb)-3xflag-myc* | This study | Fig. 1-figure supplement 1 |
| pRM472 | pTV118N | *vemP(G93amb)-3xflag-myc* | This study | Fig. 1-figure supplement 1 |
| pRM421 | pTV118N | *vemP(L94amb)-3xflag-myc* | This study | Fig. 1-figure supplement 1 |
| pRM496 | pTV118N | *vemP(D95amb)-3xflag-myc* | This study | Fig. 1-figure supplement 1 |
| pRM400 | pTV118N | *vemP(D96amb)-3xflag-myc* | This study | Fig. 1-figure supplement 1 |
| pRM449 | pTV118N | *vemP(E97amb)-3xflag-myc* | This study | Fig. 1-figure supplement 1 |
| pRM473 | pTV118N | *vemP(H98amb)-3xflag-myc* | This study | Fig. 1-figure supplement 1 |
| pRM422 | pTV118N | *vemP(V99amb)-3xflag-myc* | This study | Fig. 1-figure supplement 1 |
| pRM497 | pTV118N | *vemP(D100amb)-3xflag-myc* | This study | Fig. 1-figure supplement 1 |
| pRM401 | pTV118N | *vemP(V101amb)-3xflag-myc* | This study | Fig. 1-figure supplement 1 |
| pRM450 | pTV118N | *vemP(V102amb)-3xflag-myc* | This study | Fig. 1-figure supplement 1 |
| pRM474 | pTV118N | *vemP(G103amb)-3xflag-myc* | This study | Fig. 1-figure supplement 1 |
| pRM434 | pTV118N | *vemP(D104amb)-3xflag-myc* | This study | Fig. 1-figure supplement 1 |
| pRM498 | pTV118N | *vemP(L105amb)-3xflag-myc* | This study | Fig. 1-figure supplement 1 |
| pRM402 | pTV118N | *vemP(N106amb)-3xflag-myc* | This study | Fig. 1-figure supplement 1 |
| pRM451 | pTV118N | *vemP(T107amb)-3xflag-myc* | This study | Fig. 1-figure supplement 1 |
| pRM475 | pTV118N | *vemP(P108amb)-3xflag-myc* | This study | Fig. 1-figure supplement 1 |
| pRM423 | pTV118N | *vemP(F109amb)-3xflag-myc* | This study | Fig. 1-figure supplement 1 |
| pRM499 | pTV118N | *vemP(Y110amb)-3xflag-myc* | This study | Fig. 1-figure supplement 1 |
| pRM403 | pTV118N | *vemP(V111amb)-3xflag-myc* | This study | Fig. 1-figure supplement 1 |
| pRM452 | pTV118N | *vemP(D112amb)-3xflag-myc* | This study | Fig. 1-figure supplement 1 |
| pRM476 | pTV118N | *vemP(A113amb)-3xflag-myc* | This study | Fig. 1-figure supplement 1 |
| pRM424 | pTV118N | *vemP(G114amb)-3xflag-myc* | This study | Fig. 1-figure supplement 1 |
| pRM500 | pTV118N | *vemP(Y115amb)-3xflag-myc* | This study | Fig. 1-figure supplement 1 |
| pRM404 | pTV118N | *vemP(A116amb)-3xflag-myc* | This study | Fig. 1-figure supplement 1 |
| pRM453 | pTV118N | *vemP(Y117amb)-3xflag-myc* | This study | Fig. 1-figure supplement 1 |
| pRM485 | pTV118N | *vemP(S118amb)-3xflag-myc* | This study | Fig. 1-figure supplement 1 |
| pRM425 | pTV118N | *vemP(L119amb)-3xflag-myc* | This study | Fig. 1-figure supplement 1 |
| pRM501 | pTV118N | *vemP(M120amb)-3xflag-myc* | This study | Fig. 1-figure supplement 1 |
| pRM405 | pTV118N | *vemP(D121amb)-3xflag-myc* | This study | Fig. 1-figure supplement 1 |
| pRM454 | pTV118N | *vemP(I122amb)-3xflag-myc* | This study | Fig. 1-figure supplement 1 |
| pRM477 | pTV118N | *vemP(N123amb)-3xflag-myc* | This study | Fig. 1-figure supplement 1 |
| pRM426 | pTV118N | *vemP(W124amb)-3xflag-myc* | This study | Figs. 1B, Fig. 1-figure supplements 1, 2 |
| pRM510 | pTV118N | *vemP(R125amb)-3xflag-myc* | This study | Fig. 1-figure supplement 1 |
| pRM406 | pTV118N | *vemP(Q126amb)-3xflag-myc* | This study | Fig. 1-figure supplement 1 |
| pRM455 | pTV118N | *vemP(N127amb)-3xflag-myc* | This study | Fig. 1-figure supplement 1 |
| pRM478 | pTV118N | *vemP(Q128amb)-3xflag-myc* | This study | Fig. 1-figure supplement 1 |
| pRM427 | pTV118N | *vemP(Y129amb)-3xflag-myc* | This study | Fig. 1-figure supplement 1 |
| pRM502 | pTV118N | *vemP(T130amb)-3xflag-myc* | This study | Fig. 1-figure supplement 1 |
| pRM407 | pTV118N | *vemP(F131amb)-3xflag-myc* | This study | Fig. 1-figure supplement 1 |
| pRM456 | pTV118N | *vemP(Y132amb)-3xflag-myc* | This study | Fig. 1-figure supplement 1 |
| pRM479 | pTV118N | *vemP(H133amb)-3xflag-myc* | This study | Fig. 1-figure supplement 1 |
| pRM428 | pTV118N | *vemP(F134amb)-3xflag-myc* | This study | Fig. 1-figure supplement 1 |
| pRM511 | pTV118N | *vemP(T135amb)-3xflag-myc* | This study | Fig. 1-figure supplement 1 |
| pRM408 | pTV118N | *vemP(S136amb)-3xflag-myc* | This study | Fig. 1-figure supplement 1 |
| pRM457 | pTV118N | *vemP(D137amb)-3xflag-myc* | This study | Fig. 1-figure supplement 1 |
| pRM520 | pTV118N | *his_10_-vemP-3xflag-myc* | This study |  |
| pRM557 | pTV118N | *his_10_-vemP(*Δ*SS)-3xflag-myc* | This study | Fig. 5-figure supplement 1 |
| pRM563 | pTV118N | *his_10_-vemP(*Δ*SS, W143A)-3xflag-myc* | This study | Fig. 5-figure supplement 1 |
| pRM562 | pTV118N | *his_10_-vemP(*Δ*SS, R85W)-3xflag-myc* | This study | Fig. 5-figure supplement 1 |
| pBAD24 |  | Expression vector; P*_araBAD_*, Amp^R^ | (Guzman et al., 1995) |  |
| pHM810 | pBAD24 | *vemP-V.secD2F/2* | (Ishii et al., 2015) |  |
| pHM846 | pBAD24 | *vemP(W143A)-V.secD2/F2* | (Ishii et al., 2015) |  |
| pRM662 | pBAD24 | *vemP(R85W)-V.secD2/F2* | This study |  |
| pRM737 | pBAD24 | *V.ffh* | This study |  |
| pRM740 | pBAD24 | *V.ffh 5'-araC-*P*_ara_-ffh* | This study |  |
| pRM83 |  | Expression vector; P*_lac_*, Spc^R^ | (Miyazaki et al., 2016) |  |
| pRM83c |  | pRM83-constitutive expression plasimid | This study |  |
| pRM656 | pRM83c | *his_10_-secD/secF* | This study | Fig. 4D |
| pRM661 | pRM83c | *ppiD* | This study | Fig. 4D |
| pSTV28 |  | Expression vector; P*_lac_*, Cm^R^ | Takara Bio |  |
| pST30 | pSTV28 | Syd | (Shimoike et al., 1995) | Fig. 5C |
| pUC118 |  | Expression vector; P*_lac_*, Amp^R^ | Takara Bio |  |
| pRM570 | pUC118 | *spa-tag*::*kan* | This study |  |
| pRM573 | pUC118 | *his_10_-tag*::*kan* | This study |  |
| pRM670 | pUC118 | pUC118-*V.ppiD 5'*-*V.ppiD-V.ppiD 3'* | This study |  |
| pRM674 | pUC118 | pUC118-*V.ppiD 5'*-Δ*V.ppiD-V.ppiD 3'* | This study |  |
| pSW7848 | R6K γ-ori-based suicide vector, P*_araBAD_-ccdB*, Cm^R^ | | (Val et al., 2012) |  |
| pRM691 | pSW7848 | *V.ppiD 5'-*Δ*V.ppiD-V.ppiD 3'* | This study |  |
| pRM744 | pSW7848 | *V.ffh 5'-araC-*P*_ara_-V.ffh* | This study |  |

**(References)**

Guzman LM, Belin D, Carson MJ, Beckwith J. 1995. Tight regulation, modulation, and high-level expression by vectors containing the arabinose P_BAD_ promoter. *J Bacteriol* **177**:4121–4130. doi:10.1128/jb.177.14.4121-4130.1995

Ishii E, Chiba S, Hashimoto N, Kojima S, Homma M, Ito K, Akiyama Y, Mori H. 2015. Nascent chain-monitored remodeling of the Sec machinery for salinity adaptation of marine bacteria. *Proc Natl Acad Sci U S A* **112**:E5513-22. doi:10.1073/pnas.1513001112

Miyazaki R, Yura T, Suzuki T, Dohmae N, Mori H, Akiyama Y. 2016. A novel SRP recognition sequence in the homeostatic control region of heat shock transcription factor σ^32^. *Sci Rep* **6**:24147. doi:10.1038/srep24147

Mori H, Sakashita S, Ito J, Ishii E, Akiyama Y. 2018. Identification and characterization of a translation arrest motif in VemP by systematic mutational analysis. *J Biol Chem* **293**:2915–2926. doi:10.1074/jbc.M117.816561

Shimoike T, Taura T, Kihara A, Yoshihisa T, Akiyama Y, Cannon K, Ito K. 1995. Product of a new gene, *syd*, functionally interacts with SecY when overproduced in *Escherichia coli*. *J Biol Chem* **270**. doi:10.1074/jbc.270.10.5519

Val M-E, Skovgaard O, Ducos-Galand M, Bland MJ, Mazel D. 2012. Genome engineering in *Vibrio cholerae* a feasible approach to address biological issues. *PLoS Genet* **8**:e1002472. doi:10.1371/journal.pgen.1002472

Young TS, Ahmad I, Yin JA, Schultz PG. 2010. An enhanced system for unnatural amino acid mutagenesis in *E. coli*. *J Mol Biol* **395**:361–374. doi:10.1016/j.jmb.2009.10.030
