# Supplementary material for: Fine interaction profiling of VemP and mechanisms responsible for its translocation-coupled arrest-cancelation
Source: eLife. 2020 Dec 15;9:e62623. doi: 10.7554/eLife.62623 (PMC7793623; doi:10.7554/eLife.62623)
Supplement: Supplementary file 3. — Primers used in this study [file elife-62623-supp3.docx]

**Table S3. Primers used in this study.**

| Name | Sequence (5' to 3') |
| --- | --- |
| his_10_-vemP-f | GCGCGCCATGGCTCATCATCATCATCATCATCATCATCATCATCAGATTTGCTTGAAAAAATTGC |
| M4C | TACGCCAGCTGGCGAAAGGG |
| lacO1-c-f | GTATGTTGTGTGGAATTATTGTCGGATAACAATTTCACAC |
| lacO1-c-r | GTGTGAAATTGTTATCCGACAATAATTCCACACAACATAC |
| lacO1-c2-f | GTGTGGAATTATTGTCGGACAATAATTTCACACAGG |
| lacO1-c2-r | CCTGTGTGAAATTATTGTCCGACAATAATTCCACAC |
| lacO1-c3-f | GTGTGGAATTATTGTTAGACAATAATTTCACACAGG |
| lacO1-c3-r | CCTGTGTGAAATTATTGTCTAACAATAATTCCACAC |
| his_10_-secD-f | CGCGAAGCTTAGGAGGTTTAAATTATGCATCATCATCATCATCATCATCATCATCATTTAAACCGTTATCCTTTGTGG |
|  |  |
| secF-r | CGCGGTCGACTTACGGCAGAATTGACGG |
| ppiD-f | CGCGAAGCTTAGGAGGTTTAAATTATGATGGACAGCTTACGCAC |
| ppiD-r | CGCGGTCGACTTATTGCTGTTCCAGCGCATC |
| Va-ppiD-f | CGGTACCCGGGGATCCCAAAAGATGGCCCAAGTGC |
| Va-ppiD-r | ATGCCTGCAGGTCGACTAGGGATGAACCTGGCAGG |
| del-Va-ppiD-f | GCACAATTAGTGTAATTTGCATG |
| del-Va-ppiD-r | TTACACTAATTGTGCAATTTAACTGCTCTCCACAATTG |
| pSW-Va-ppiD-f | TCTGCGAGGCTGGCCCAAAAGATGGCCCAAGTGC |
| pSW-Va-ppiD-r | GATACCGTCGACGCCTAGGGATGAACCTGGCAGG |
| Va-ffh-f | GAGGAATTCACCATGTTTGAGAATTTAACGGATCG |
| Va-ffh-r | AAACAGCCAAGCTTGCATGCTTAACGACCAAATGGGTTGAAG |
| u-ffh-f | TTGAGTGAGCTGATAAAGCGACTGCCAAAATACGC |
| u-ffh-r | AACATGAGCAGATCCGTCTCTTCGCTAAAATGGCG |
| pRM737-f | GGATCTGCTCATGTTTGACAG |
| pRM737-r | TATCAGCTCACTCAAAGGCG |
| pSW-Va-ffh-f | TCTGCGAGGCTGGCCAAGCGACTGCCAAAATACGC |
| pSW-Va-ffh-r | GATACCGTCGACGCCCTTAACGACCAAATGGGTTG |
| spa-kan-f | CGCGCGGAATTCGATGATGAGAAGCATGAAGGG |
| spa-kan-r | CGCGCGGTCGACTGAGGATGACGAGCACATCC |
| his_10_-kan-f | CGCGGAATTCCACCATCACCACCATCACCATCATCATCATTAATGACCTGAGGGGAGATATC |
| kan-Para-f | ACAGCTTATCATCGACATATGAATATCCTCCTTAG |
| kan-Para-r | TTGACAGGCACATTATGACCTGAGGGGAGATATC |
| ffh-his_10_-f | ATGAGAAGCATGAAGGGTATGATGCCCCCAGGCTTCCCTGGTCGCCACCATCACCACCATCACC |
| ffh-his_10_-r | TGACGAGCACATCCCGGTGCCAAAATGGCAAACAAGCCAGGCCGACATATGAATATCCTCCTTAG |
| secY-his_10_-f | GGTCTGGAAAAACGTGGCCTGCATAGCCGCGAGAAGAAAAAATCCCACCATCACCACCATCACC |
| secY-his_10_-r | GCACGAACTTTCATTTTTACTCTCCGTAACTTCTCGGGCGACCAACATATGAATATCCTCCTTAG |
| yidC-his_10_-f | GGTCTGGAAAAACGTGGCCTGCATAGCCGCGAGAAGAAAAAATCCCACCATCACCACCATCACC |
| yidC-his_10_-r | AAGGCGGTCAACTGACCGCCCTTATTTTAGCGAAAACTCACCGAACATATGAATATCCTCCTTAG |
| ppiD-his_10_-f | CGTAAAGAGGCGAAAATCAAAATTGGCGATGCGCTGGAACAGCAACACCATCACCACCATCACC |
| ppiD-his_10_-r | TCTTGTTACAGCTGCAACGCATTTTTTTGCGAGGCGGCTTCAGGACATATGAATATCCTCCTTAG |
